# Oculomotor response inhibition and distractor suppression following prior self-control exertion in athletes

**DOI:** 10.3389/fpsyg.2026.1928706

**Published:** 2026-09-25

**Authors:** Maoyan Fan, Junyi Zhou, Wenxin Xu

**Affiliations:** School of Physical Education and Sport Science, Fujian Normal University, Fuzhou, China

**Keywords:** antisaccade, athletes, distractor suppression, ego depletion, eye tracking, oculomotor response inhibition, self-control, visual search

## Abstract

**Introduction:**

Prior self-control exertion can affect subsequent attention and performance, but the specific visual control processes involved in athletes remain unclear. The present study examined oculomotor response inhibition and distractor suppression during visual search following prior self-control exertion in athletes.

**Methods:**

In Experiment 1 (*N* = 75), prior self-control exertion was manipulated using a proportion-congruent Stroop task, and oculomotor response inhibition was assessed using prosaccade and antisaccade tasks. In Experiment 2 (*N* = 62), participants completed visual search under no-activation and activation conditions after either the depletion or the no-depletion Stroop condition.

**Results:**

In Experiment 1, the high-depletion group showed longer antisaccade latencies and greater inhibitory control costs, while prosaccade latencies and saccade error rates did not differ significantly across groups. In Experiment 2, within the depletion group, the activation condition produced longer reaction times, delayed first fixation on the target, more fixations, and longer cumulative fixation duration on distractors than the no-activation condition.

**Discussion:**

Together, the findings indicate that prior self-control exertion was associated with latency-based costs in oculomotor response inhibition and greater difficulty suppressing salient distractors during visual search in this athlete sample.

## 1 Introduction

During daily training and competition, athletes often need to maintain self-control to regulate emotion, cope with pressure, and suppress task-irrelevant distractions when they are fatigued (Alder et al., 2019; Englert et al., 2020). With persistent self-control demands and a lack of recovery, athletes are more likely to become impulsive and distracted, thereby decreasing their performance at critical moments (Giboin and Wolff, 2019). Self-control is the deliberate effort to exert control over oneself by attempting to change how one would otherwise think, feel, or behave (Muraven and Baumeister, 2000). This capacity has often been conceptualized as vulnerable to prior exertion; repeated or prolonged exertion may induce ego depletion, making subsequent acts of self-control harder to sustain (Baumeister et al., 2007; Dang, 2018; Vohs et al., 2021). In this framework, ego depletion can temporarily impair subsequent performance (Baumeister et al., 2007; Muraven and Baumeister, 2000). However, the effects of ego depletion on subsequent self-control and the mechanisms underlying these effects remain under debate (Dang, 2018; Friese et al., 2019; Etherton et al., 2018).

The strength model proposes that acts of self-control consume self-control resources, which in turn can impair performance on subsequent self-control tasks (Muraven and Baumeister, 2000; Baumeister et al., 2007). In contrast, the process model explains such aftereffects as shifts in motivation and attention rather than depletion of a unitary resource. It proposes reduced processing of control-relevant cues and less willingness to invest further self-control (Berkman et al., 2017; Inzlicht and Schmeichel, 2012). Regardless of the proposed mechanism, evidence for ego depletion effects remains inconsistent, and observed effects may vary with the characteristics and self-control demands of both the initial and subsequent tasks (Hagger et al., 2016; Dang, 2018; Friese et al., 2019; Vohs et al., 2021). In sequential-task paradigms, depletion-related effects may therefore be more detectable when the subsequent task places greater demands on inhibitory control, although this pattern is not consistent across tasks and studies (Radel et al., 2019; Singh and Göritz, 2018).

Inhibitory control is a core executive function. It helps regulate attention and behavior by suppressing impulses and distractions (Diamond, 2013). It can involve suppressing a prepotent response or resisting interference from irrelevant information (Nigg, 2000; Friedman and Miyake, 2004). Eye movements provide observable measures of visual selection and response control (Kowler, 2011; Spering, 2022). Eye tracking is widely used in sport research to examine how athletes select visual information for action (Mann et al., 2007; Silva et al., 2022). However, visual demands and eye movement patterns differ across sports and task contexts (Mann et al., 2007; Silva et al., 2022). These differences make it important to examine specific visual control processes in athletes rather than treat visual control as a single ability. The present study therefore examined oculomotor response inhibition and distractor suppression during visual search in an athlete sample.

The prosaccade and antisaccade tasks provide a direct comparison of oculomotor response inhibition. Both tasks require a saccadic response to a peripheral target, but they differ in inhibitory control demands. In prosaccade trials, participants look toward a peripheral target. This response is largely reflexive and requires relatively little inhibitory control. In antisaccade trials, participants must suppress the reflexive saccade toward the target and look in the opposite direction. This response requires greater oculomotor inhibition (Everling et al., 1998; Munoz and Everling, 2004). The two tasks share similar basic visual and saccadic requirements. This comparison can therefore show whether prior self-control exertion affects saccadic performance generally or has a greater effect when inhibition is required. If prior self-control exertion causes a general decline, both tasks should be affected. If the effect depends on inhibitory control, the cost should be greater in antisaccade trials.

Visual search requires the selection of task-relevant information among competing stimuli (Corbetta and Shulman, 2002; Theeuwes, 2010; Awh et al., 2012). Salient distractors can capture attention (Sawaki and Luck, 2010; Gaspar and McDonald, 2014; Gaspelin and Luck, 2018). Goal-directed control can reduce their interference (Sawaki and Luck, 2010; Gaspar and McDonald, 2014; Gaspelin and Luck, 2018). Many competitive sport situations also require visual search (Mann et al., 2007; Brimmell et al., 2024; Silva et al., 2022). Athletes often need to identify task-relevant information among multiple visual cues and resist interference from irrelevant stimuli (Mann et al., 2007; Brimmell et al., 2024; Silva et al., 2022). Sport research suggests that prior self-control exertion can impair attention regulation and decision performance when athletes must resist distraction (Furley et al., 2013; Englert, 2017; Boat et al., 2021). If prior self-control exertion weakens distractor suppression, its effects should be more evident when salient distractors are present.

Previous sport research has reported changes in attention and performance after prior self-control exertion, but the specific visual control processes involved remain unclear (Furley et al., 2013; Englert et al., 2015, 2019; Boat et al., 2021). Experiment 1 examined oculomotor response inhibition by comparing prosaccade and antisaccade performance. Experiment 2 examined distractor suppression during visual search by comparing conditions with and without a salient distractor. Together, the two experiments examined whether depletion-related costs emerge in oculomotor response inhibition, distractor suppression, or both. We hypothesized that depletion-related costs would be greater in antisaccade than prosaccade performance in Experiment 1 and greater in the activation condition than in the no-activation condition in Experiment 2.

## 2 Experiment 1: oculomotor response inhibition following prior self-control exertion

### 2.1 Methods

#### 2.1.1 Participants

An *a priori* power analysis was conducted using G*Power for a mixed-design ANOVA, focusing on the within-between interaction. The parameters were set as follows: effect size f = 0.25, α = 0.05, power = 0.95, number of groups = 3, number of measurements = 2, correlation among repeated measures = 0.50, and non-sphericity correction ε = 1. The analysis indicated that a minimum total sample size of 66 participants was required, corresponding to approximately 22 participants per group. The final analytic sample included 75 participants, with 25 participants assigned to each group. A total of 75 undergraduate students majoring in athletic training at a university in China were recruited as participants (39 females; mean age = 20.08 years, SD = 1.79). All participants were certified athletes registered within the Chinese National Athlete Classification System and reported normal or corrected-to-normal vision, no major medical or neurological conditions, and no limitations in physical activity. Participants received course credit for their participation. Written informed consent was obtained from all participants prior to participation.

#### 2.1.2 Procedure

Upon arrival at the laboratory, participants received standardized instructions and were randomly assigned to one of three groups: no-depletion (*N* = 25), low-depletion (*N* = 25), or high-depletion (*N* = 25). They first completed the Trait Self-Control Scale (SCS) to assess individual differences in self-control. They then performed the Stroop task. Immediately afterward, they completed a manipulation check scale and the Brief Mood Introspection Scale (BMIS) to assess perceived effort and current mood. Subsequently, participants were asked to perform a saccadic eye movement task that included both prosaccade and antisaccade trials. At the end of the experiment, all participants were informed of the purpose of this experiment.

#### 2.1.3 Materials

The present study employed the sequential-task paradigm derived from the strength model of self-control, consisting of an initial self-control exertion task and a subsequent probe task (Baumeister et al., 1998). In Experiment 1, the Stroop task was used as the initial self-control exertion task because it requires participants to suppress word meaning and respond to ink color, thereby imposing demands on inhibitory control (MacLeod, 1991). This task has been widely used in research on ego depletion and prior self-control exertion (Hagger et al., 2010; Dang et al., 2021). In the present manipulation, the high-depletion and low-depletion groups were distinguished by varying the proportion of congruent and incongruent trials in the Stroop task. The probe task was an oculomotor task, which included both prosaccade and antisaccade trials. Prior evidence indicates that antisaccade performance is a reliable index of inhibitory control (Munoz and Everling, 2004). Accordingly, prosaccade trials were used as the baseline condition, whereas antisaccade trials served as the primary condition for assessing oculomotor response inhibition. Both the Stroop and oculomotor tasks were programmed using SR Research Experiment Builder, and stimuli were presented on a 17-inch Dell Vostro laptop computer (resolution: 1,920 × 1,080; refresh rate 150 Hz). In the oculomotor task, black dots (RGB: 0, 0, 0) were displayed on a gray background (RGB: 153, 153, 153). In the Stroop task, colored words were presented on a white background (RGB: 255, 255, 255) in red (RGB: 255, 0, 0), yellow (RGB: 255, 192, 0), blue (RGB: 0, 176, 240), or green (RGB: 0, 176, 80). During the experiment, participants viewed the display binocularly and were instructed to maintain fixation on the screen. Eye movements were recorded using the EyeLink Portable Duo system with a sampling rate of 1000 Hz. The right eye was selected *a priori* as the recorded and analyzed eye and was used consistently across all participants, following the monocular recording approach used in previous EyeLink pro-/antisaccade studies (Bey et al., 2018; Jin et al., 2022). In addition, participants completed three self-report scales to assess potential individual differences in self-control, perceived task demands, and mood: (1) Trait self-control was assessed using the 19-item SCS with a five-point Likert response format (1 = strongly disagree, 5 = strongly agree). Higher scores on this scale indicate higher trait self-control (Tangney et al., 2004). (2) A two-item manipulation check assessed the perceived difficulty and effort of the Stroop task on seven-point scales (1 = very easy, 7 = very difficult). These ratings were used to verify whether the Stroop manipulation increased subjective task demands, rather than to directly measure ego depletion states (Dang et al., 2021). (3) Momentary mood was measured with the BMIS (16 items; four-point response format: 1 = definitely do not feel, 4 = definitely feel) (Mayer and Gaschke, 1988).

#### 2.1.4 Stroop task

The Stroop task (Figure 1a) was used to manipulate prior self-control exertion. The task consisted of four types of stimuli, which were Chinese translations of the words red, green, blue, and yellow. Each word was displayed on a computer screen in any of these four ink colors. Participants were instructed to react as fast as possible in response to the color of the ink of the word, without paying attention to the meaning of the word. They responded by pressing the key corresponding to the ink color (“D” for red, “F” for yellow, “J” for blue, and “K” for green). Each trial began with a central fixation cross presented for 1,000 ms, followed by the presentation of a stimulus word. The stimulus remained on the screen until a response was made. While the meaning of the word and the color of the ink were the same in congruent trials, they differed in incongruent trials. Participants in the high-depletion group completed 120 trials with a higher proportion of incongruent trials (72 incongruent and 48 congruent), whereas participants in the low-depletion group completed 120 trials with a lower proportion of incongruent trials (48 incongruent and 72 congruent). In the no-depletion group, all 120 trials were congruent. Before the formal task, participants completed eight practice trials (four congruent and four incongruent in the depletion groups; eight congruent in the no-depletion group).

**FIGURE 1:**
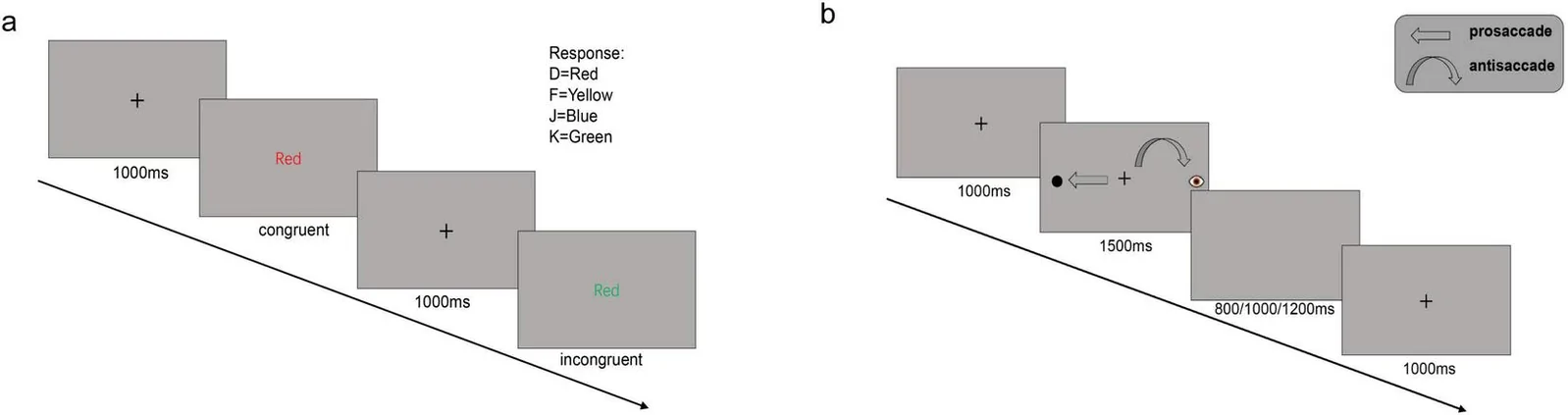
Procedures of the Stroop task and probe task. **(a)** Stroop task procedure and response mapping. **(b)** Prosaccade and antisaccade task procedure.

#### 2.1.5 Probe task

The probe task consisted of prosaccade and antisaccade conditions. Each condition comprised five practice trials and 60 experimental trials (65 trials in total). Each trial began with a central fixation cross presented for 1,000 ms, followed by a target stimulus, shown as a black circle (1.2° × 1.2°), which appeared randomly on either the left or right side of the screen, with 30 trials presented on each side. The target remained on the screen for 1,500 ms. The intertrial interval consisted of a blank screen presented for 800, 1,000, or 1,200 ms in random order. In the prosaccade condition, participants were instructed to move their eyes as quickly as possible toward the target location when it appeared, reflecting relatively low inhibitory control demands. In the antisaccade condition, participants were instructed to suppress the reflexive saccade toward the target and instead generate a saccade to the mirror location of the target stimulus, which placed greater demands on inhibitory control (Figure 1b).

#### 2.1.6 Statistical analyses

Data Viewer software (SR Research, Toronto, Ontario, Canada) was used to analyze the raw eye movement data. To ensure that the included eye movement data met the predefined criteria, the following criteria for inclusion were used in the analyses (Jazbec et al., 2006): (1) saccades with a latency between 80 and 800 ms; (2) saccade amplitude greater than 3°; and (3) saccade duration greater than or equal to 25 ms. Based on these criteria, the following saccade indices were derived: (1) saccade error rate, defined as the probability that a participant executed an incorrect saccadic response. Specifically, an antisaccade error was coded when the first saccade after target onset was directed toward the target rather than the mirror location, whereas a prosaccade error was coded when the first saccade was directed toward the mirror location rather than toward the target; (2) antisaccade latency, defined as the time elapsed from target onset to the onset of the first saccade toward the mirror image location of the target; (3) prosaccade latency, defined as the time elapsed from target onset to the onset of the first saccade toward the target location; (4) inhibitory control cost, defined as the difference between antisaccade latency and prosaccade latency (antisaccade minus prosaccade latency), with larger values indicating greater latency-based inhibitory control costs. All statistical analyses were conducted in R (version 4.4.3). All tests were two-tailed with an alpha level of 0.05. Before inferential analyses, data were screened to ensure that the assumptions of the applied statistical models were reasonably met.

The SCS and BMIS were used only as baseline and group equivalence checks and were not entered as covariates in the primary analyses. These measures, together with manipulation check ratings of perceived difficulty and effort, were analyzed separately using one-way ANOVAs with Group as the between-subjects factor. Saccade error rates and saccadic latencies were analyzed using mixed-design ANOVAs with Group (no-depletion, low-depletion, high-depletion) as the between-subjects factor and Saccade Task (prosaccade, antisaccade) as the within-subjects factor. Inhibitory control cost was analyzed using a one-way ANOVA with Group as the between-subjects factor. Significant interactions were followed by simple effects analyses, and significant omnibus effects were followed by planned pairwise comparisons, as appropriate. Effect sizes are reported as partial η^2^_*p*_ for ANOVA effects. Descriptive statistics are presented as means and standard deviations.

### 2.2 Results

#### 2.2.1 Stroop task performance

As expected, Stroop task performance shows significant group differences. The main effect of group is significant, *F*(2, 72) = 34.854, *p* < 0.001, η*^2^_*p*_* = 0.492. Pairwise comparisons show that the high-depletion group (*M* = 1044.83 ms, SD = 236.52) responds more slowly than the low-depletion group (M = 916.33 ms, SD = 146.14), *t*(72) = 2.595, *p* < 0.05. The low-depletion group also responds more slowly than the no-depletion group (*M* = 640.32 ms, SD = 120.93), *t*(72) = 5.575, *p* < 0.001. Reaction times in the high-depletion group are likewise longer than in the no-depletion group, *t*(72) = 8.170, *p* < 0.001. These results indicate that the Stroop manipulation produced graded differences in task performance across groups, consistent with the intended manipulation of prior self-control exertion.

#### 2.2.2 Saccade error rate

Saccade error rate shows a significant main effect of task type (Table 1). Error rates are higher in antisaccade trials (10.77%) than in prosaccade trials (0.35%). The main effect of task type is significant, *F*(1, 72) = 86.560, *p* < 0.001, η*^2^_*p*_* = 0.546. In contrast, the main effect of group is not significant, *F*(2, 72) = 0.748, *p* > 0.05, η*^2^_*p*_* = 0.020, and the interaction between group and task type is also not significant, *F*(2, 72) = 0.689, *p* > 0.05, η*^2^_*p*_* = 0.019.

**TABLE 1:** Eye movement measures in prosaccade and antisaccade trials (M ± SD).

Measure	No-depletion group	Low-depletion group	High-depletion group
Prosaccade error rate (%)	0.47 ± 1.70	0.30 ± 0.91	0.29 ± 0.69
Antisaccade error rate (%)	9.64 ± 8.79	9.97 ± 9.89	12.69 ± 10.81
Prosaccade latency (ms)	161.30 ± 23.86	168.84 ± 42.71	163.72 ± 22.84
Antisaccade latency (ms)	206.71 ± 28.05	227.10 ± 38.90	249.79 ± 38.57
Inhibitory control cost (ms)	45.41 ± 33.94	58.26 ± 48.23	86.07 ± 35.20

#### 2.2.3 Saccade latency

Saccade latency shows a significant main effect of task type (Figure 2a and Table 1). Saccade latency is longer in antisaccade trials (227.87 ms) than in prosaccade trials (164.61 ms), *F*(1, 72) = 190.53, *p* < 0.001, η^2^_*p*_ = 0.726. The main effect of group is also significant, *F*(2, 72) = 4.55, *p* < 0.05, η^2^_*p*_ = 0.112. Pairwise comparisons show that saccade latency in the high-depletion group (206.75 ms) is longer than that in the no-depletion group (184.01 ms), *t*(72) = 2.990, *p* < 0.05. The interaction between group and task type is significant, *F*(2, 72) = 6.860, *p* < 0.001, η^2^_*p*_ = 0.160. Simple effects analyses show that, in prosaccade trials, saccade latency does not differ significantly across groups, *F*(2, 72) = 0.381, *p* > 0.05, η^2^_*p*_ = 0.010. In contrast, in antisaccade trials, saccade latency differs significantly across groups, *F*(2, 72) = 9.20, *p* < 0.001, η^2^_*p*_ = 0.203. Pairwise comparisons show that antisaccade latency is longer in the high-depletion group (249.79 ms) than in the no-depletion group (206.71 ms), *t*(72) = 4.286, *p* < 0.001. Antisaccade latency in the low-depletion group (227.10 ms) does not differ significantly from that in the no-depletion group, *t*(72) = 2.029, *p* > 0.05, or from that in the high-depletion group, *t*(72) = 2.257, *p* > 0.05.

**FIGURE 2:**
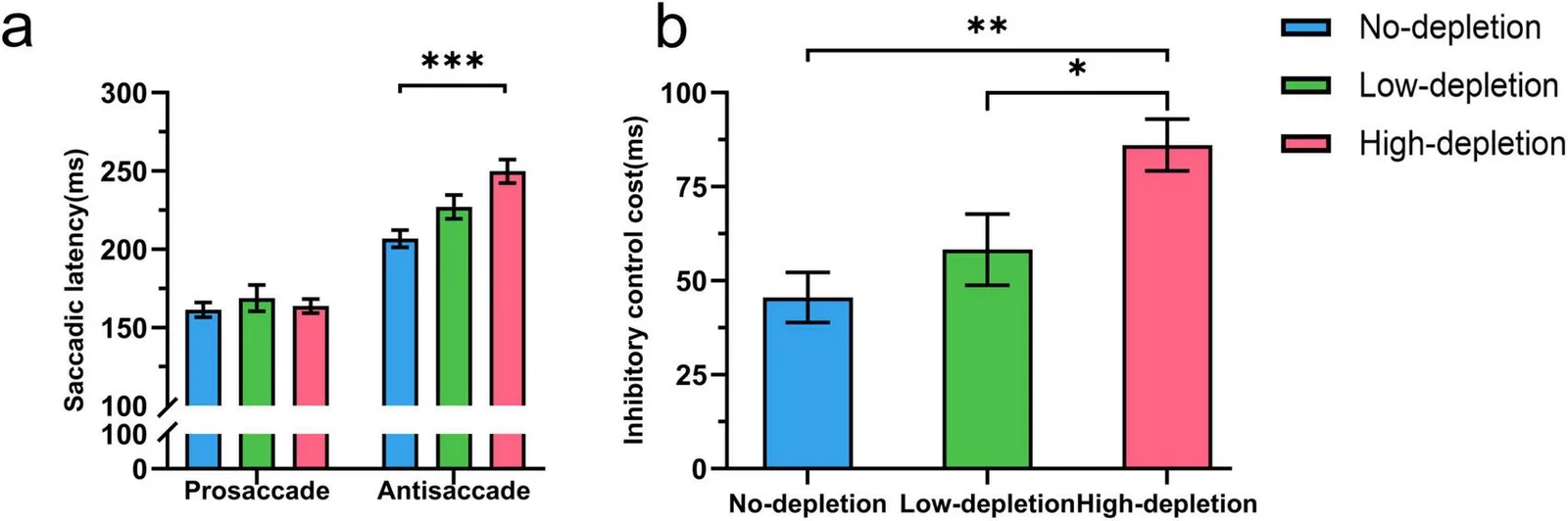
Saccadic latency and inhibitory control cost across depletion conditions. **(a)** Mean saccadic latency (ms) for prosaccade and antisaccade trials; note the break in the y-axis. **(b)** Inhibitory control cost (ms) across groups. Error bars represent the standard error of the mean (SEM). Asterisks denote significant pairwise differences *p < 0.05; **p < 0.01; ***p < 0.001.

#### 2.2.4 Inhibitory control cost

Inhibitory control cost differs significantly across groups (Figure 2b and Table 1). The main effect of group is significant, *F*(2, 72) = 6.857, *p* < 0.01, η*^2^_*p*_* = 0.160. Pairwise comparisons show that the high-depletion group (*M* = 86.07 ms, SD = 35.20) exhibits larger inhibitory control costs than the no-depletion group (*M* = 45.41 ms, SD = 33.94), *t*(72) = 3.623, *p* < 0.01, and also larger costs than the low-depletion group (*M* = 58.26 ms, SD = 48.23), *t*(72) = 2.477, *p* < 0.05.

#### 2.2.5 Baseline and manipulation checks

Self-Control Scale scores did not differ significantly across groups, *F*(2, 72) = 0.946, *p* > 0.05, η^2^_*p*_ = 0.026, indicating no baseline group difference in trait self-control. The SCS showed high internal consistency (Cronbach’s α = 0.906). To evaluate whether the Stroop manipulation increased subjective task demands, scores on self-report measures of perceived difficulty and effort were examined. For perceived difficulty, the main effect of group was significant, *F*(2, 72) = 143.589, *p* < 0.001, η^2^_*p*_ = 0.800. Pairwise comparisons showed that the high-depletion group (*M* = 4.36, SD = 0.76) reported higher perceived difficulty than the low-depletion group (*M* = 2.52, SD = 0.51), *t*(72) = 10.432, *p* < 0.001. The no-depletion group (*M* = 1.40, SD = 0.58) reported lower perceived difficulty than both the low-depletion group, *t*(72) = 6.350, *p* < 0.001, and the high-depletion group, *t*(72) = 16.782, *p* < 0.001.

A similar pattern emerged for effort. The main effect of group was significant, *F*(2, 72) = 69.825, *p* < 0.001, η^2^_*p*_ = 0.660. The high-depletion group (*M* = 4.16, SD = 0.75) reported greater effort than the low-depletion group (*M* = 3.00, SD = 0.71), *t*(72) = 5.616, *p* < 0.001, whereas the no-depletion group (*M* = 1.72, SD = 0.74) reported lower effort than both the low-depletion group, *t*(72) = 6.197, *p* < 0.001, and the high-depletion group, *t*(72) = 11.813, *p* < 0.001. The manipulation check scale showed high internal consistency (Cronbach’s α = 0.876). Finally, BMIS scores showed no significant group effect, *F*(2, 72) = 0.368, *p* > 0.05, η^2^_*p*_ = 0.010, indicating that the Stroop manipulation did not differentially influence mood state. The BMIS showed high internal consistency (Cronbach’s α = 0.911).

### 2.3 Discussion

Experiment 1 examined whether prior self-control exertion affected oculomotor response inhibition in athletes. Participants in the high-depletion group showed longer antisaccade latencies and greater inhibitory control costs than those in the no-depletion group. Prosaccade latencies and saccade error rates did not differ significantly across groups. This pattern indicates that the depletion-related effect was concentrated in latency-based measures of oculomotor response inhibition. These findings support the first hypothesis and suggest that prior self-control exertion primarily slowed inhibition-dependent oculomotor responses in this task.

## 3 Experiment 2: distractor suppression during visual search following prior self-control exertion

Experiment 1 showed that prior self-control exertion mainly affected latency-based measures of oculomotor response inhibition. Experiment 2 examined distractor suppression during visual search. Participants searched for a target among competing stimuli under conditions with and without a salient task-irrelevant distractor. This design tested whether prior self-control exertion increased distractor interference during visual selection.

### 3.1 Methods

#### 3.1.1 Participants

An *a priori* power analysis was conducted using G*Power for a mixed-design ANOVA, focusing on the within-between interaction. The parameters were set as follows: effect size f = 0.25, α = 0.05, power = 0.95, number of groups = 2, number of measurements = 2, correlation among repeated measures = 0.50, and non-sphericity correction ε = 1. The analysis indicated that a minimum total sample size of 54 participants was required, corresponding to approximately 27 participants per group. A total of 62 undergraduate students majoring in athletic training at a university in China were recruited as participants (31 females; mean age = 19.96 years, SD = 1.68). All participants met the same inclusion criteria as in Experiment 1. Participants received course credit for their participation. Written informed consent was obtained from all participants prior to participation.

#### 3.1.2 Procedure

Upon arrival at the laboratory, participants received standardized instructions and were randomly assigned to the depletion group or the no-depletion group (*n* = 31 per group). They first completed the SCS to assess individual differences in self-control. They then performed the Stroop task. Immediately afterward, they completed a manipulation check scale and the BMIS to assess perceived effort and current mood. Subsequently, participants performed a visual search task to assess distractor suppression under conditions with and without a salient distractor. At the end of the experiment, all participants were informed of the purpose of this experiment.

#### 3.1.3 Stroop task

In Experiment 2, prior self-control exertion was manipulated using the same Stroop task as in Experiment 1 (see Figure 1a). Participants in the depletion group completed 120 trials with a higher proportion of incongruent trials (72 incongruent and 48 congruent), whereas participants in the no-depletion group completed 120 congruent trials.

#### 3.1.4 Probe task

The probe task consisted of a no-activation condition and an activation condition. Eye movements during the visual search task were recorded using the same EyeLink system and right-eye recording approach described in Experiment 1. Each condition comprised 10 practice trials and 96 experimental trials (106 trials in total). Each trial began with a central fixation cross presented for 1,000 ms, followed by the presentation of a search array consisting of eight ball-type stimuli (soccer ball, tennis ball, rubber ball, golf ball, volleyball, softball, basketball, and bowling ball) arranged in a circular layout around the center of the screen, with positions randomized and evenly distributed. All stimuli were presented in achromatic colors (black, white, and shades of gray). Participants were instructed to search for the target stimulus (soccer ball) and indicate its presence or absence by pressing the “J” key if the target was present and the “F” key if it was absent. The search array remained on the screen until a response was made. Each trial ended with a gray intertrial interval of randomly varying duration (700, 800, or 900 ms). In the no-activation condition, the search array contained no salient distractor (Figure 3a). In the activation condition, the procedure was identical except that a salient distractor was included: one non-target stimulus was randomly selected and displayed in red, whereas all other stimuli remained achromatic (Figure 3b). In both conditions, 50% of the experimental trials contained the target stimulus, with the remaining seven stimuli randomly selected from the ball types; in the remaining 50% of trials, the target was absent. The activation condition therefore required participants to suppress interference from a salient distractor during target search. The trial procedure and response mapping are illustrated in Figure 3c.

**FIGURE 3:**
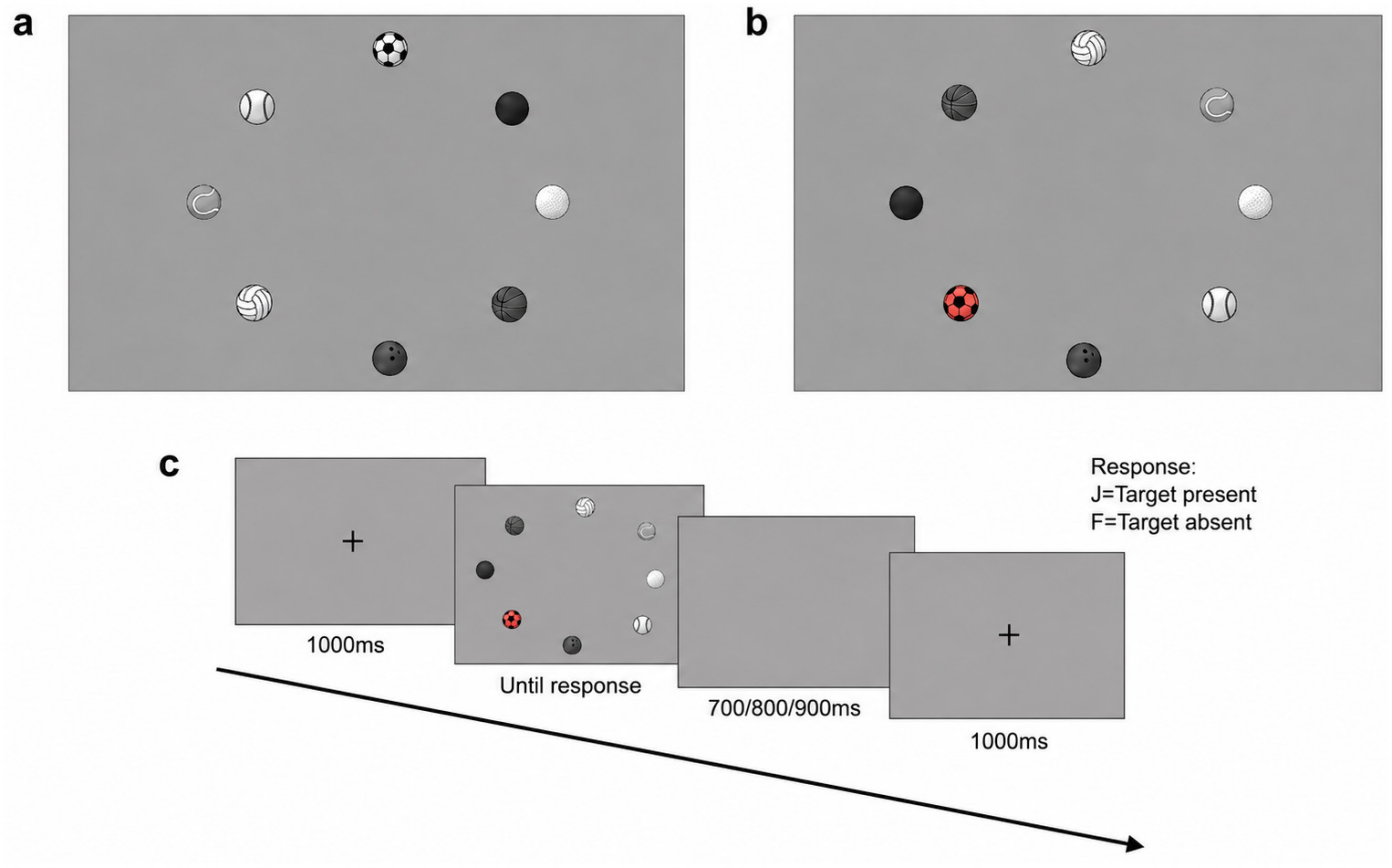
Visual search task stimuli, procedure, and response mapping. **(a)** No-activation display. **(b)** Activation display with a salient distractor. **(c)** Visual search task procedure and response mapping.

#### 3.1.5 Statistical analyses

Data Viewer software (SR Research, Toronto, Ontario, Canada) was used to analyze the raw eye movement data. The following behavioral and eye movement measures were defined and extracted for analysis: (1) visual search error rate, defined as the proportion of trials in which participants made an incorrect judgment about target presence or absence; (2) elapsed time to first fixation on target, defined as the time elapsed from the onset of the search display to the onset of the first fixation on the target; (3) visual search reaction time, defined as the time elapsed from the onset of the search display to the participant’s manual response indicating target presence or absence; (4) number of fixations, defined as the total number of fixations recorded from the onset of the search display to the onset of the blank screen; (5) cumulative fixation duration on distractors, defined as the summed duration of all fixation events falling within non-target distractor areas of interest across the 48 target-present formal trials within each search condition. Each distractor area of interest included the distractor object and a surrounding region extending 1.8° of visual angle; (6) first-pass duration on the target, defined as the sum of fixation durations from the initial entry of gaze on the target until the first exit from the target within a valid trial; and (7) fixation duration on the target, defined as the total fixation time on the target from the onset of the search display to the end of the trial.

All statistical analyses were conducted in R version 4.4.3 using RStudio (version 2024.12.1 + 563). All tests were two-tailed with an alpha level of 0.05. The SCS and BMIS were used only as baseline and group equivalence checks and were not entered as covariates in the primary analyses. These measures, together with manipulation check ratings of perceived difficulty and effort, were analyzed separately using one-way ANOVAs with Group as the between-subjects factor. Behavioral and eye movement outcomes were analyzed at the participant-by-condition level. Trial-level observations were first averaged within each search condition for each participant, and the resulting condition means were submitted to mixed-design ANOVAs with Group (no-depletion, depletion) as the between-subjects factor and Search Condition (no-activation, activation) as the within-subjects factor. Significant interactions were followed by simple effects analyses, and significant omnibus effects were followed by planned pairwise comparisons, as appropriate. Effect sizes are reported as partial η^2^_*p*_ for ANOVA effects. Descriptive statistics are presented as means and standard deviations.

### 3.2 Results

#### 3.2.1 Stroop task performance

Stroop task performance shows a significant group difference. The main effect of group is significant, *F*(1, 60) = 47.443, *p* < 0.001, η*^2^_*p*_* = 0.442. Pairwise comparison indicates that the depletion group (*M* = 985.86 ms) responds more slowly than the no-depletion group (*M* = 645.01 ms), *t*(60) = 6.888, *p* < 0.001. These results indicate that the Stroop manipulation produced the expected group difference in task performance, consistent with the intended manipulation of prior self-control exertion.

#### 3.2.2 Visual search error rate

Visual search error rates show a significant main effect of activation condition (Table 2). Error rates are higher in the activation condition (1.18%) than in the no-activation condition (0.71%). The main effect of activation condition is significant, *F*(1, 60) = 5.073, *p* < 0.05, η*^2^_*p*_* = 0.078. The main effect of group is not significant, *F*(1, 60) = 1.126, *p* > 0.05, η*^2^_*p*_* = 0.018. The Group × Activation Condition interaction did not reach statistical significance, although the numerical pattern was in the expected direction, *F*(1, 60) = 3.132, *p* = 0.080, η*^2^_*p*_* = 0.050. Exploratory simple effects analyses suggested that error rates did not differ between the no-activation and activation conditions in the no-depletion group, *F*(1, 60) = 0.116, *p* > 0.05, η*^2^_*p*_* = 0.002, whereas the depletion group showed a higher error rate in the activation condition (1.48%) than in the no-activation condition (0.64%), *F*(1, 60) = 8.089, *p* < 0.01, η*^2^_*p*_* = 0.119. Because the interaction did not reach conventional statistical significance, this error rate pattern should be interpreted cautiously and was not treated as primary evidence for depletion-related visual search costs.

**TABLE 2:** Behavioral performance in the visual search task (M ± SD).

Measure	No-depletion group	Depletion group
Error rate in no-activation condition (%)	0.77 ± 1.11	0.64 ± 0.84
Error rate in activation condition (%)	0.87 ± 1.18	1.48 ± 1.56
Reaction time in the no-activation condition (ms)	664.41 ± 171.42	680.88 ± 231.01
Reaction time in the activation condition (ms)	738.41 ± 203.80	1025.83 ± 430.28

#### 3.2.3 Visual search reaction time

Visual search reaction times show significant effects of activation condition and group (Figure 4a and Table 2). Reaction times are longer in the activation condition (*M* = 882.12 ms) than in the no-activation condition (*M* = 672.65 ms). The main effect of activation condition is significant, *F*(1, 60) = 41.590, *p* < 0.001, η*^2^_*p*_* = 0.409. The main effect of group is also significant, *F*(1, 60) = 5.870, *p* < 0.05, η*^2^_*p*_* = 0.089; participants in the depletion group (*M* = 853.36 ms) respond more slowly overall than those in the no-depletion group (*M* = 701.41 ms), *t*(60) = 2.422, *p* < 0.05. The interaction between group and activation condition is significant, *F*(1, 60) = 17.400, *p* < 0.001, η*^2^_*p*_* = 0.225. Simple effects analyses show that, in the no-depletion group, reaction times do not differ significantly between the no-activation and activation conditions, *F*(1, 60) = 2.595, *p* > 0.05, η*^2^_*p*_* = 0.041. In contrast, in the depletion group, the activation condition exerts a pronounced effect, *F*(1, 60) = 56.400, *p* < 0.001, η^2^_*p*_ = 0.485. Reaction times are longer in the activation condition (*M* = 1025.83 ms) than in the no-activation condition (*M* = 680.88 ms), *t*(60) = 7.510, *p* < 0.001.

**FIGURE 4:**
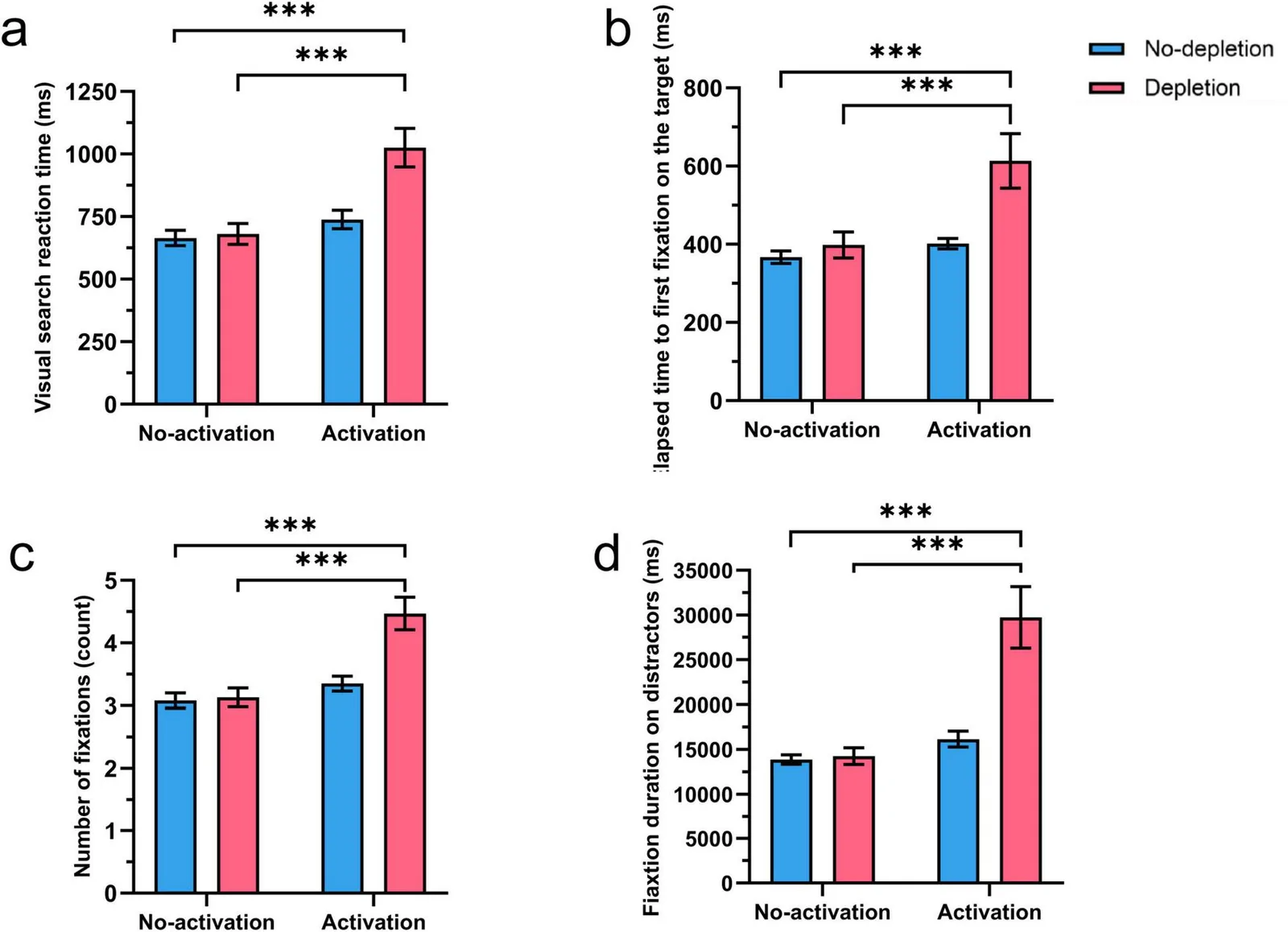
Visual search performance and eye-tracking indices as a function of activation condition and group. **(a)** Visual search reaction time (ms). **(b)** Elapsed time to first fixation on target (ms). **(c)** Number of fixations (count). **(d)** Cumulative fixation duration on distractors (ms). Error bars represent the standard error of the mean (SEM). Asterisks denote significant pairwise differences ***p < 0.001.

#### 3.2.4 Elapsed time to first fixation on target

Elapsed time to first fixation on target shows significant effects of activation condition and group (Figure 4b and Table 3). Elapsed time is longer in the activation condition (*M* = 507.44 ms) than in the no-activation condition (*M* = 382.65 ms). The main effect of activation condition is significant, *F*(1, 60) = 15.980, *p* < 0.001, η^2^_*p*_ = 0.210. The main effect of group is also significant, *F*(1, 60) = 6.700, *p* < 0.05, η^2^_*p*_ = 0.100; elapsed time to first fixation is longer in the depletion group (*M* = 505.79 ms) than in the no-depletion group (*M* = 384.30 ms), *t*(60) = 2.589, *p* < 0.05. The interaction between group and activation condition is significant, *F*(1, 60) = 8.380, *p* < 0.01, η^2^_*p*_ = 0.123. Simple effects analyses show that, in the no-depletion group, the activation condition does not significantly affect elapsed time to first fixation, *F*(1, 60) = 0.608, *p* > 0.05, η^2^_*p*_ = 0.010. In contrast, in the depletion group, the activation condition exerts a pronounced effect, *F*(1, 60) = 23.760, *p* < 0.001, η^2^_*p*_ = 0.284. Elapsed time to first fixation is longer in the activation condition (*M* = 613.36 ms) than in the no-activation condition (*M* = 398.21 ms), *t*(60) = 4.874, *p* < 0.001.

**TABLE 3:** Eye-tracking measures in the visual search task (M ± SD).

Measure	No-depletion group	Depletion group
Elapsed time to first fixation on target (no-activation) (ms)	367.09 ± 87.85	398.21 ± 184.48
Elapsed time to first fixation on target (activation) (ms)	401.51 ± 74.23	613.36 ± 386.92
Number of fixations (no-activation)	3.08 ± 0.67	3.13 ± 0.83
Number of fixations (activation)	3.35 ± 0.69	4.47 ± 1.45
Cumulative fixation duration on distractors (no-activation) (ms)	13859.74 ± 2910.36	14242.16 ± 5150.06
Cumulative fixation duration on distractors (activation) (ms)	16155.35 ± 4959.02	29724.84 ± 19153.10
First-pass duration on target (no-activation) (ms)	177.65 ± 46.72	183.04 ± 34.88
First-pass duration on target (activation) (ms)	186.78 ± 37.99	185.67 ± 30.85
Fixation duration on target (no-activation) (ms)	227.01 ± 100.35	241.70 ± 140.78
Fixation duration on target (activation) (ms)	251.02 ± 105.43	339.02 ± 175.67

#### 3.2.5 Number of fixations

The number of fixations shows significant effects of activation condition and group in the visual search task (Figure 4c and Table 3). The number of fixations is higher in the activation condition than in the no-activation condition (*M* = 3.91 and *M* = 3.10, respectively). The main effect of activation condition is significant, *F*(1, 60) = 37.700, *p* < 0.001, η*^2^_*p*_* = 0.386. The main effect of group is also significant, *F*(1, 60) = 8.000, *p* < 0.01, η*^2^_*p*_* = 0.118; the number of fixations is higher in the depletion group (*M* = 3.80) than in the no-depletion group (*M* = 3.22), *t*(60) = 2.828, *p* < 0.01. The interaction between group and activation condition is significant, *F*(1, 60) = 16.860, *p* < 0.001, η*^2^_*p*_* = 0.219. Simple effects analyses show that, in the no-depletion group, the activation condition does not significantly affect the number of fixations, *F*(1, 60) = 2.069, *p* > 0.05, η*^2^_*p*_* = 0.033. In contrast, in the depletion group, the activation condition exerts a clear effect, *F*(1, 60) = 15.170, *p* < 0.001, η^2^_*p*_ = 0.202. The number of fixations is higher in the activation condition (*M* = 4.47) than in the no-activation condition (*M* = 3.13), *t*(60) = 3.895, *p* < 0.001.

#### 3.2.6 Cumulative fixation duration on distractors

Cumulative fixation duration on distractors shows significant effects of activation condition and group in the visual search task (Figure 4d and Table 3). Cumulative fixation duration on distractors is longer in the activation condition (*M* = 22940.10 ms) than in the no-activation condition (*M* = 14050.95 ms). The main effect of activation condition is significant, *F*(1, 60) = 27.171, *p* < 0.001, η*^2^_*p*_* = 0.312. The main effect of group is also significant, *F*(1, 60) = 12.259, *p* < 0.001, η*^2^_*p*_* = 0.170; cumulative fixation duration on distractors is longer in the depletion group (*M* = 21983.50 ms) than in the no-depletion group (*M* = 15007.55 ms), *t*(60) = 3.501, *p* < 0.001. The interaction between group and activation condition is significant, *F*(1, 60) = 14.949, *p* < 0.001, η*^2^_*p*_* = 0.199. Simple effects analyses show that, in the no-depletion group, the activation condition does not significantly affect cumulative fixation duration on distractors, *F*(1, 60) = 0.906, *p* > 0.05, η*^2^_*p*_* = 0.015. In contrast, in the depletion group, the activation condition exerts a pronounced effect, *F*(1, 60) = 41.214, *p* < 0.001, η*^2^_*p*_* = 0.407. Cumulative fixation duration on distractors is longer in the activation condition (*M* = 29724.84 ms) than in the no-activation condition (*M* = 14242.16 ms), *t*(60) = 6.420, *p* < 0.001.

#### 3.2.7 First-pass duration on target

First-pass duration on target was not significantly affected by activation condition or group (Table 3). The main effect of activation condition was not significant, *F*(1, 60) = 1.192, *p* > 0.05, η^2^_*p*_ = 0.019, and the main effect of group was also not significant, *F*(1, 60) = 0.071, *p* > 0.05, η^2^_*p*_ = 0.001. The interaction between group and activation condition was likewise not significant, *F*(1, 60) = 0.365, *p* > 0.05, η^2^_*p*_ = 0.006. These results indicate that initial viewing duration on the target did not differ significantly by activation condition or group.

#### 3.2.8 Fixation duration on target

Fixation duration on target shows significant effects of activation condition and a significant interaction between group and activation condition in the visual search task (Table 3). Fixation duration on the target is longer in the activation condition than in the no-activation condition (*M* = 295.02 ms and *M* = 234.35 ms, respectively). The main effect of activation condition is significant, *F*(1, 60) = 17.368, *p* < 0.001, η*^2^_*p*_* = 0.224. The main effect of group is not significant, *F*(1, 60) = 2.783, *p* > 0.05, η*^2^_*p*_* = 0.044. The interaction between group and activation condition is significant, *F*(1, 60) = 6.340, *p* < 0.05, η*^2^_*p*_* = 0.096. Simple effects analyses show that, in the no-depletion group, the activation condition does not significantly affect fixation duration on target, *F*(1, 60) = 1.361, *p* > 0.05, η*^2^_*p*_* = 0.022. In contrast, in the depletion group, the activation condition exerts a clear effect, *F*(1, 60) = 22.347, *p* < 0.001, η*^2^_*p*_* = 0.271. Fixation duration on target is longer in the activation condition (*M* = 339.02 ms) than in the no-activation condition (*M* = 241.70 ms), *t*(60) = 4.727, *p* < 0.001.

#### 3.2.9 Baseline and manipulation checks

Self-Control Scale scores did not differ significantly between the depletion and no-depletion groups, *F*(1, 60) = 0.523, *p* > 0.05, η^2^_*p*_ = 0.009, indicating no baseline group difference in trait self-control. The SCS showed high internal consistency (Cronbach’s α = 0.918). To evaluate whether the Stroop manipulation increased subjective task demands, scores on self-report measures of perceived difficulty and effort were examined. For perceived difficulty, the main effect of group was significant, *F*(1, 60) = 266.304, *p* < 0.001, η^2^_*p*_ = 0.816. Pairwise comparison showed that the depletion group (*M* = 3.90, SD = 0.54) reported higher perceived difficulty than the no-depletion group (*M* = 1.65, SD = 0.55), *t*(60) = 16.319, *p* < 0.001.

A similar pattern emerged for effort. The main effect of group was significant, *F*(1, 60) = 188.976, *p* < 0.001, η*^2^_*p*_* = 0.759. The depletion group (*M* = 4.29, SD = 0.82) reported greater effort than the no-depletion group (*M* = 1.71, SD = 0.64), *t*(60) = 13.747, *p* < 0.001. Together with the perceived difficulty results, these findings indicate that the Stroop manipulation increased subjective task demands in the depletion group. The manipulation check scale showed high internal consistency (Cronbach’s α = 0.904). Finally, BMIS scores showed no significant group effect, *F*(1, 60) = 0.058, *p* > 0.05, η*^2^_*p*_* = 0.001, indicating that the Stroop manipulation did not differentially influence mood state. The BMIS showed high internal consistency (Cronbach’s α = 0.930).

### 3.3 Discussion

Experiment 2 examined whether prior self-control exertion affected distractor suppression during visual search. The results show that depletion-related differences were more evident when a salient distractor was present. In the depletion group, the activation condition was associated with slower target identification, more fixations, and longer cumulative fixation duration on distractors than the no-activation condition. These condition differences were not significant in the no-depletion group. Manipulation checks again showed higher subjective task demands in the depletion group. The groups did not differ in trait self-control or mood. These findings support the second hypothesis that depletion-related costs would be greater when salient distractors were present. However, the pattern was not consistent across all visual search measures. Overall, the results suggest that prior self-control exertion was associated with greater difficulty suppressing salient distractors during visual search rather than a uniform decline in visual search performance.

## 4 General discussion

Across the two experiments, we examined how prior self-control exertion affected oculomotor response inhibition and distractor suppression in athletes. Experiment 1 showed that prior self-control exertion mainly affected latency-based measures of oculomotor response inhibition. The high-depletion group showed longer antisaccade latencies and greater inhibitory control costs, while prosaccade latencies and saccade error rates did not differ significantly across groups. Experiment 2 showed that depletion-related differences were more evident during visual search when a salient distractor was present. Within the depletion group, the activation condition produced longer reaction times, delayed first fixation on the target, more fixations, and longer cumulative fixation duration on distractors than the no-activation condition. Together, these findings show that the effects of prior self-control exertion were not uniform across visual control measures. These effects were observed in oculomotor response inhibition in Experiment 1 and distractor suppression during visual search in Experiment 2.

### 4.1 Oculomotor response inhibition and distractor suppression following prior self-control exertion

Experiment 1 provides evidence that prior self-control exertion affected the speed of oculomotor response inhibition rather than saccadic performance generally. Antisaccade trials require suppression of the reflexive saccade toward a peripheral target before a voluntary saccade can be generated in the opposite direction (Everling et al., 1998; Munoz and Everling, 2004). The longer antisaccade latencies and greater inhibitory control costs in the high-depletion group therefore indicate a greater temporal cost when this prepotent response had to be suppressed. The absence of significant group differences in prosaccade latency and saccade error rate further suggests that the observed effect was not a general slowing of saccadic responding or a broad loss of response accuracy.

Experiment 2 examined distractor suppression during visual search. Salient distractors can capture attention and delay selection of task-relevant information, whereas goal-directed control can reduce such interference (Sawaki and Luck, 2010; Gaspar and McDonald, 2014; Gaspelin and Luck, 2018). In the depletion group, the presence of a salient distractor was associated with slower responses, delayed first fixation on the target, more fixations, and longer cumulative fixation duration on distractors. These changes indicate less efficient visual selection in the presence of competing salient information. However, the effect did not appear across all visual search measures, which argues against a uniform deterioration of visual search performance.

Taken together, the two experiments refine previous sport research showing that prior self-control exertion can alter subsequent attentional and performance outcomes (Furley et al., 2013; Englert et al., 2015, 2019; Boat et al., 2021). The present findings locate these changes more specifically in two visual control processes. Experiment 1 identified a latency-based cost in oculomotor response inhibition, whereas Experiment 2 identified changes in visual selection when salient distractors competed with the target. The present study therefore provides process-level evidence by identifying where depletion-related effects were expressed within visually guided control in the examined athlete sample.

### 4.2 Practical implications for sports performance and training

The practical relevance of these findings is likely to depend on the visual demands of the sport and the specific performance situation. Sports differ in the extent to which athletes must monitor multiple visual cues, rapidly select task-relevant information, and resist interference from salient but irrelevant stimuli (Mann et al., 2007; Brimmell et al., 2024). The present findings may therefore be more relevant to situations in which successful performance depends on rapid visual selection and suppression of competing information. They should not be assumed to apply equally across sports with different visual and attentional demands.

From a training perspective, the findings suggest that accumulated self-control demands may be worth considering when athletes subsequently perform tasks that require rapid response inhibition or resistance to visual distraction. Coaches and practitioners may need to consider the timing of cognitively demanding activities when these visual control processes are important for subsequent performance. However, the present experiments did not test whether specific cognitive training methods can reduce these effects. Recommendations regarding antisaccade training, visual search training, or other neurocognitive interventions therefore require direct empirical evaluation.

Accordingly, the main practical implication is not a universal training prescription but greater attention to prior self-control demands in sport situations that rely heavily on rapid visual selection and suppression of distraction.

### 4.3 Limitations and future directions

Several limitations warrant consideration. Although the final analytic sample exceeded the minimum sample size estimated by the *a priori* power analysis, the per-group sample size was still modest. Therefore, the present study may have had limited sensitivity for detecting smaller effects, particularly interaction effects. Future studies with larger samples are needed to further verify the robustness of the present findings. The sample characteristics also limit the external validity of the findings. Participants were undergraduate students majoring in athletic training and were not stratified by sport discipline or competitive level. Different sports place different demands on visual search, visual tracking, and the suppression of irrelevant information (Mann et al., 2007; Brimmell et al., 2024). The present study therefore cannot determine whether the observed effects have the same magnitude or pattern across sports with different visual and attentional demands. Future studies should directly compare athletes from sports with different perceptual and visual control requirements.

A further limitation concerns the interpretation of the Stroop manipulation and the mechanisms underlying the observed effects. Although the manipulation checks confirmed differences in perceived difficulty and effort, these measures reflected subjective task demands rather than ego depletion itself. Accordingly, alternative explanations involving increased cognitive load or non-specific affective and motivational states, such as boredom, frustration, or disengagement, cannot be fully ruled out. The present study also relied primarily on behavioral and oculomotor indices. The absence of complementary physiological or neural measures limits the extent to which the mechanisms underlying the observed behavioral and eye movement effects can be identified. Future research could address these issues by incorporating load-matched control conditions, basic affective and motivational assessments, and convergent physiological or neural indicators alongside behavioral and eye movement measures. Such approaches would help distinguish the effects of prior self-control exertion from alternative explanations and clarify the conditions under which these effects emerge.

## 5 Conclusion

The present findings show that prior self-control exertion was associated with specific changes in visual control in the examined athlete sample. These changes were expressed as latency-based costs in oculomotor response inhibition and greater difficulty suppressing salient distractors during visual search.

## Data Availability

The original contributions presented in the study are publicly available. This data can be found here: https://doi.org/10.5281/zenodo.22840400.
